# A Molecular Phylogeny of the Nightjar Louse Fly *Pseudolynchia garzettae* (Rondani, 1979) (Diptera: Hippoboscidae) with a Diagnostic Morphological Description of a Sequenced Specimen

**DOI:** 10.1007/s11686-026-01248-7

**Published:** 2026-04-06

**Authors:** Denise C. Wawman, Steven R. Fiddaman, Abigail S. Bailey, Adrian L. Smith

**Affiliations:** 1 Edward Grey Institute of Field Ornithology, Department of Biology, University of Oxford, South Parks Road, Oxford, OX1 3RB UK; 2 Department of Biology, University of Oxford, South Parks Road, Oxford, OX1 3RB UK; 3 The Pirbright Institute, Ash Rd, Pirbright, Woking, GU24 0NF UK; 4 The John Krebs Field Station, Department of Biology, University of Oxford, Wytham, Oxford, OX2 8QJ UK

**Keywords:** Sequencing, Hippoboscidae, Louse fly, Ectoparasite, DNA barcoding

## Abstract

**Background:**

Cytochrome oxidase subunit I (COX1) DNA sequences are widely used as a means of identifying Eukaryotic species, but in some taxonomic groups, such as the louse flies (Diptera: Hippoboscidae), there are many species that have not been sequenced. Until very recently (Wawman, 2025), *Pseudolynchia garzettae* (Rondani, 1897) had no sequences in either of the two main databases, NCBI GenBank and International Barcode of Life v3 (BOLD), but there are multiple sequences identified only to the level of the genus *Pseudolynchia*.

**Methods:**

A Nightjar Louse Fly *Pseudolynchia garzettae*, was taken from an adult male European Nightjar *Caprimulgus europaeus* (Linnaeus, 1758), in Wales, United Kingdom, identified on the basis of its morphology and sequenced. The sequences were compared to all of the high-quality published COXI sequences for the genus *Pseudolynchia*.

**Results:**

A 662 bp mitochondrial COX1 DNA sequence was obtained, confirming the sequenced fly as a separate species to all of the other sequenced specimens, which were all a single species *Pseudolynchia canariensis *(Macquart, 1839). A detailed morphological description of the fly and a differential diagnosis table is provided to aid researchers working with species in this genus.

**Conclusions:**

The DNA sequence supports the current taxonomy that places *P. garzettae* in the genus *Pseudolynchia* with *P. canariensis*, within the same clade as the genus *Icosta*.

**Graphical Abstract:**

**Figure:**
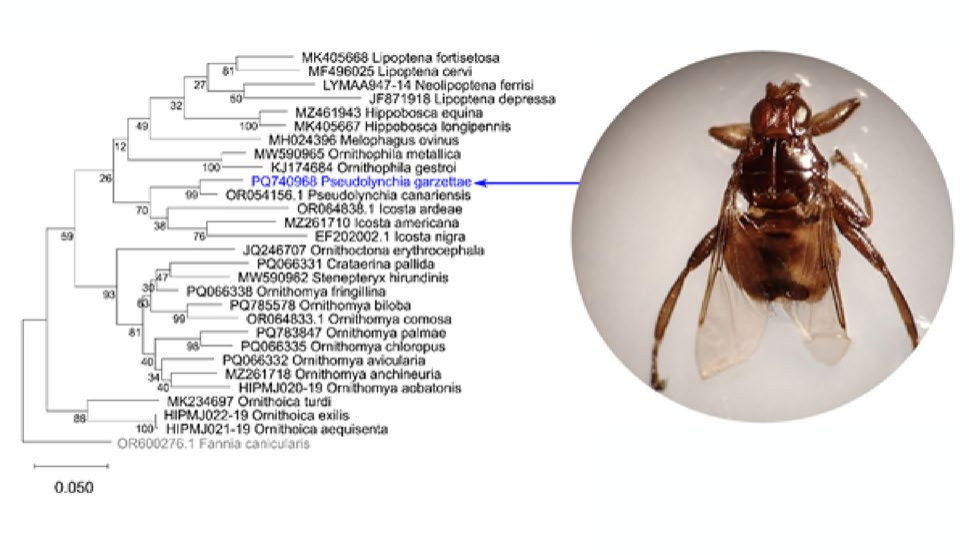
• Until recently, *Pseudolynchia garzettae* (Rondani 1897) had no previously published sequences in either of the two main databases, NCBI GenBank and International Barcode of Life v3 (BOLD) • We present a 662 base pair mitochondrial COX1 DNA sequence for the Nightjar Louse Fly *Pseudolynchia garzettae*, from a female, taken from an adult male European Nightjar *Caprimulgus europaeus* (Linnaeus, 1758), in Wales, United Kingdom. • A detailed morphological description of the sequenced fly is given to aid researchers working with species in this genus.

## Introduction

Short sequences of DNA are frequently used as “DNA barcodes” to confirm the identify of species. In Eukaryotic species the mitochondrial cytochrome c oxidase subunit I (COX1) gene sequence is widely used as it has sufficient interspecies variation to be a useful target for separating closely related species [13]. DNA sequencing can have major advantages over the traditional methods, using keys and field guides, in that it does not require expertise in species identification and may identify species or cryptic species that are not present in local field-guides, including invasive species. However, to be useful there needs to be a comprehensive library of DNA sequences for comparison [16], that do not conflict with morphological classifications [28].

The Hippoboscidae is a family of haematophagous ectoparasites that contains some species that are known vectors of trypanosomes in mammals and birds [3, 14, 37] and *Haemoproteus* spp. in birds [2, 3, 6]. Some species of Hippoboscidae have been found to contain many other pathogenic microbes, without conclusive proof that they are acting as vectors [5]. There are over 200 species within this family, many of which are of uncertain status [8], but many, especially those found in the southern hemisphere, have no published DNA sequences.

According to the most recent lists of the Hippoboscidae [8, 10], the genus *Pseudolynchia* (Bequaert, 1926) contains five species: *P. garzettae* (Rondani, 1879) [33], *P. serratipes* Maa 1966, [20], *P. brunnea* (Latreille, 1811), [17], *P. canariensis* (Macquart, 1839) [26, 27], and *P. mistula* Maa, 1969 [8]. One additional species was described while this paper was in the late stages of preparation: *Pseudolynchia maai* Lee & Obona, 2025 from Singapore was identified from DNA sequencing [19]. Of the original five species, only one, *P. canariensis*, has published COX1 sequences in NCBI GenBank (https://www.ncbi.nlm.nih.gov, last accessed 1st January 2025) and the International Barcode of Life v3 (BOLD) (https://v3.boldsystems.org, last accessed 1st January 2025), however, within the specimens listed for the genus *Pseudolynchia*, there are some that are only identified to the level of genus.

Originally described from Northern Italy [33], the Nightjar Louse Fly, *Pseudolynchia garzettae*, is a species of central and east Africa and parts of Asia, although it has been found on migrant birds in the United Kingdom (UK), France, Switzerland, Cyprus, Ukraine and Russia [11, 21, 29, 32]. In 2022, three females, two of which were gravid, were found in Wales, UK, suggesting that they might have established a breeding population [38]. *Pseudolynchia garzettae* is usually found on nightjars (Caprimulgidae) and owls (Strigidae) [11, 20], but has been found on various other species including Spotted Thick-knee (Cape Dikkop) *Burhinus capensis* (Lichtenstein, 1823), Burchell’s coucal *Centropus burchellii* (Swainson, 1838), *Pied Crow Corvus albus *(Müller, 1776), and European Swift *Apus apus* (Linnaeus, 1758) [11]. Apart from its range and a list of host species, little else is known about the ecology of this species, but in the early 1960s, Maa and Kuo examined 32 Savanna Nightjars *Caprimulgus affinis* Horsfield, 1821, captured in Taiwan, and found an infestation rate of 43.8%, with a maximum of 4 flies per host (mean 2.4 flies) [24].

We present a sequence for the Nightjar Louse Fly *Pseudolynchia garzettae*, from a vouchered specimen with a detailed description of the fly, to enable future researchers to quickly review the specimen should conflicts occur with other COXI sequences from other Hippoboscids. We compare the sequence to other sequences from the genus *Pseudolynchia*, including published sequences that have only been identified to the level of the genus, with the aim of identifying these specimens, and present a summary table of morphological methods of distinguishing the species in the genus, which do not rely on DNA sequences, subjective comparisons with other species, or genital dissection. We also aim to resolve some of the issues relating to the terminology used to describe the external genitalia of female Hippoboscidae.

## Materials and Methods

### Study Area and Material Collection

The louse flies were collected by British Trust for Ornithology (BTO) licensed bird ringers as part of the Mapping the UK’s Flat Flies Project, which has previously been described [38]. One of the ringing groups taking part, Mid-Wales Ringing Group, has a long-running study of Nightjars *Caprimulgus europaeus* (Linnaeus, 1758) in mid-Wales, and collected three female Hippoboscids of the same species, from three individual Nightjars in 2022, including two gravid females.

The fly used for sequencing was taken from an adult male Nightjar, from the Clocaenog Forest, in Wales, United Kingdom, Latitude 53.03 N, 3.49 W, at an altitude of 390 m above sea level, on 11th July 2022.

The flies were identified, using a binocular microscope and a key [15], and a key to the genus *Pseudolynchia* with descriptions of these species [20].

A selected specimen, identified morphologically as *Pseudolynchia garzettae*, was photographed using a Leica M165 C microscope with a Leica DFC490 camera mount at Oxford University Museum of Natural History. The fly was not dissected, but additional images were taken of the external genitalia using an Olympus Tough TG6 camera held over one eyepiece of a Swift S306S stereo microscope.

## Molecular Analysis

The left middle leg of a fly identified as *Pseudolynchia garzettae* was removed and kept separately in an individually labelled tube, in 70% ethanol. Upon reaching the laboratory, it was stored in a freezer at -80 °C, until it could be sequenced. The leg was disrupted under liquid nitrogen and DNA was extracted using the Qiagen Blood and Tissue kit, as per the manufacturer’s instructions.

The COXI gene fragments were amplified by PCR using Q5 High-Fidelity polymerase (New England Biolabs) with approximately 10ng input genomic DNA, according to the manufacturer’s instructions. The primers were LEPF1 ATT CAA CCA ATC ATA AAG ATA TTG G and LEPR1 TAA ACT TCT GGA TGT CCA AAA AAT CA (Integrated DNA Technologies, Belgium) [12] at the manufacturer-recommended concentration of 0.5 μm, on an Applied Biosystems Veriti PCR machine, using the following cycling conditions, 5 min at 98 °C, 40 × (30 s at 98 °C; 30 s at 60 °C; 30 s at 72 °C) then 7 min at 72 °C.

PCR products were visualised after separation on a 1% agarose gel, purified using the Qiagen PCR Clean up kit according to the manufacturer’s instructions, and then Sanger sequenced by Source BioScience, UK, using the same primers as for amplification.

## Phylogenetic Analysis

The sequence was analysed using BLASTN [1] on the GenBank website (https://www.ncbi.nlm.nih.gov/genbank, last accessed 1st January 2025), to compare it against other published DNA sequences, using the standard settings, optimised for “Somewhat similar sequences (blastn)” (https://blast.ncbi.nlm.nih.gov/Blast.cgi, last accessed 1st January 2025).

A search was performed in NCBI Genbank for all the Hippoboscidae species listed in the checklist [8], after exclusion of the fossil species *Ornithomya rottensis* (Statz, 1940) and species listed as *incertae sedis*, the available sequences were recorded, and all of the available COXI sequences were downloaded as FASTA files.

Sequences were obtained from the GenBank and BoldSystems websites, and aligned, trimmed and analysed in MEGA version 12 [16]. The analysis was run on five computing threads, for 1000 replicates, using the standard bootstrapping setting to produce a maximum likelihood tree with bootstrapped confidence limits [9], using a nearest neighbour interchange (NNI) and uniform rates in the Tamura-Nei model [36] of nucleotide substitutions. A COX1 sequence from a fly from another family of Diptera *Fannia canicularis* (Linnaeus 1761) was used as an outgroup.

## Results

### Morphological Description

#### Systematics

Order: Diptera (Linnaeus, 1758).

Family: Hippoboscidae (Samouelle, 1819).

Genus: *Pseudolynchia* Bequaert, 1926.

Species: *Pseudolynchia garzettae*, Rondani, 1879.

≡ *Olfersia garzettae*, Rondani, 1879.

= *Pseudolynchia fradeorum* Tendeiro, 1951.

## Summary

The sequenced fly was identified a female *Pseudolynchia garzettae*.

The genus was determined on the basis of the fly having fully developed wings with only one cross-vein (rm), no ocelli, and the presence of finger-like processes along the posterior edge of the scutellum [4, 20, 22, 23]. The other fully-winged louse flies in the United Kingdom, the *Ornithomya* spp. have three cross-veins. Possible vagrant species in the genera *Icosta* and *Olfersia* have two crossveins (rm and im).

It was distinguishable from *P. canariensis* because the scutellum was approximately three times as wide as it was long with a slightly curved posterior edge: *P. canariensis* has a scutellum approximately four times as wide as it is long, with a straight posterior edge. It had fewer than twenty dark setae on the dorsal thorax (*P. canariensis* has more than twenty pale setae on the dorsal thorax) and a ratio of the length to the width of the scutellum of 1:3 (it is 1:4 in *P. canariensis*) [4, 20, 22, 23]. The other species in the genus are highly unlikely to be recorded in the region [19, 22, 30] but were also excluded with reference to their descriptions.

## Diagnostic Description of the Sequenced specimen, with Comparisons To Earlier Descriptions

**Fig. 1:**
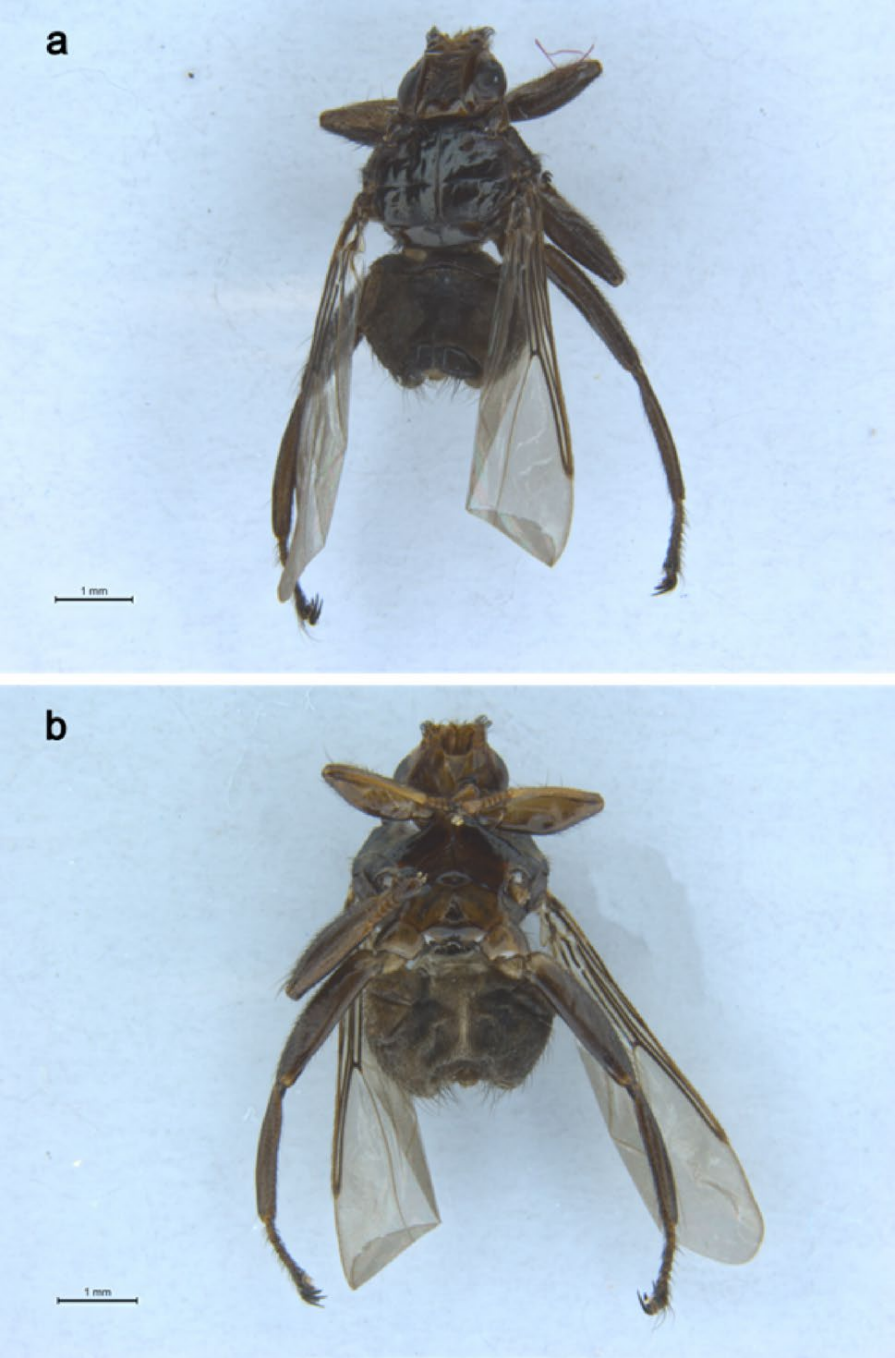
Photographs of the specimen of female *Pseudolynchia garzettae* that was sequenced.**a**, dorsal habitus;**b**, ventral habitus.

The sequenced specimen of *Pseudolynchia garzettae* is dark coloured, almost black, but paler underneath (Fig. 1a, b), as described by Rondani [33]. It is covered with black setae (bristles) but some of these have faded while the fly was stored in ethanol. The left middle leg has been removed for sequencing, leaving only part of the coxa.

It has a wing length of 5.2 mm. The other measurements are shown in in Table 1.

**Table 1:** Measurements of the specimen of *Pseudolynchia garzettae* that was sequenced

	Length (in mm)	Maximum width (in mm).
Head	1.2	1.7
Thorax	2.2	2.7
Abdomen	1.5	1.7

**Fig. 2:**
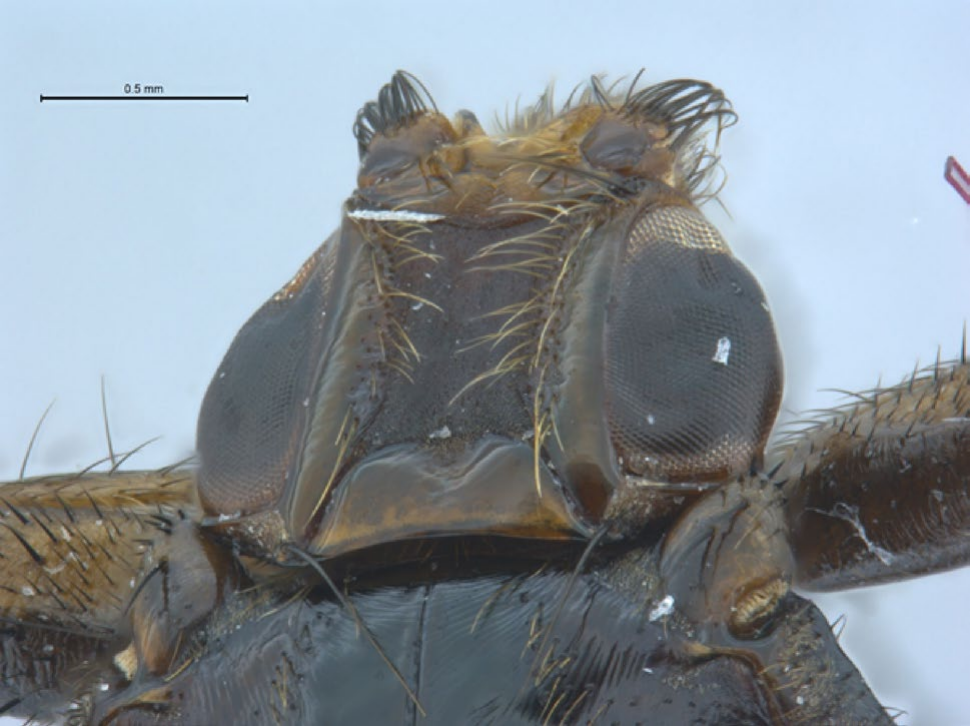
Photograph of the dorsal head of the specimen of *Pseudolynchia garzettae* that was sequenced

Head: (Fig. 2) The interocular distance, measured at the front of the head, is 1.76 times the width of the eye, which is in the range of 1.7–2 times that of the eye described by Maa [20]. Palpus short and broad, approximately as long as the lunule plus the frons (measured along median line). Vibrissal process conical and strongly projecting.

There are no ocelli.

**Fig. 3:**
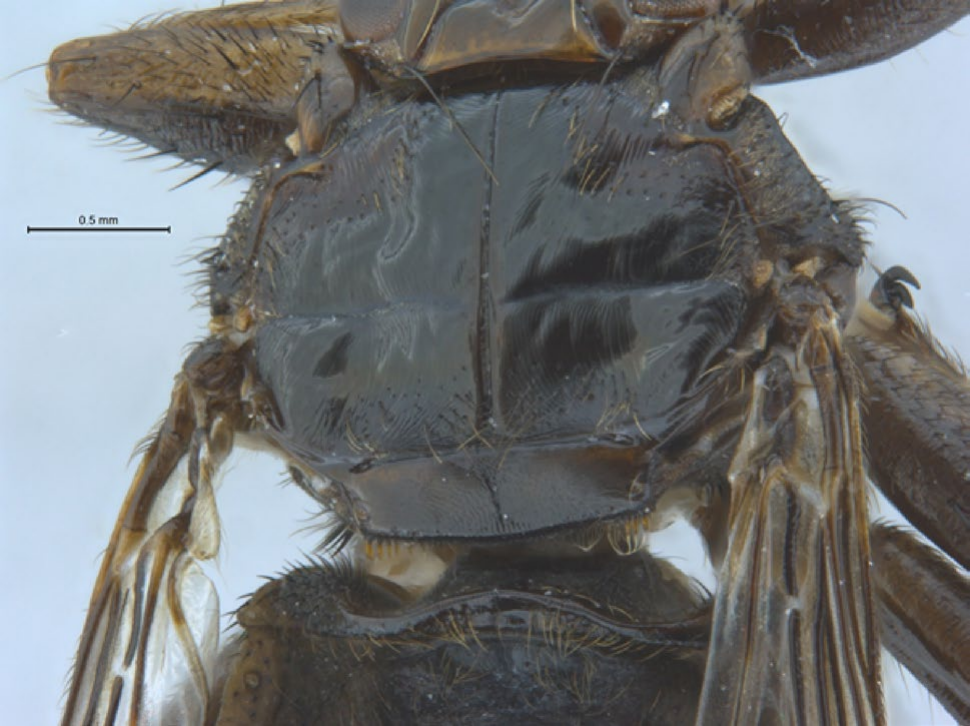
Photograph of the dorsal thorax of the specimen of *Pseudolynchia garzettae* that was sequenced

Thorax: (Fig. 3). The prescutum and scutum of the thorax are divided in half by a deep median notal suture, which continues more shallowly across the scutellum.

Humeral callus (postpronotum) conical with six short black setae along the anterior edge which point anteriorly, and two longer setae plus several shorter ones on the dorsal surface which point upwards.

Prescutum: There is a single curved tract of 14 strong, long black setae on each side of the prescutum (these have faded due to being in ethanol in the period between collection and the fly being photographed). Maa reported 13–18 long, robust setae [20]. Additionally, there are two tracts of weaker dark setae, one running almost parallel with the stronger ones, the others more laterally. The distance between basal punctures of long setae markedly more than diameter of the punctures.

Mesoscutum: The mesoscutum is divided from the prescutum by a shallow transverse mesonotal suture. There are short, dark setae across the posterior half of the mesoscutum.

Scutellum: the distance between the bases of the scutellar setae is three times the height of the scutellum. The posterior margin is slightly curved and has finger-like processes that are slightly thicker than the scutellar setae. The more medial processes are shorter and thinner than the lateral ones.

**Fig. 4:**
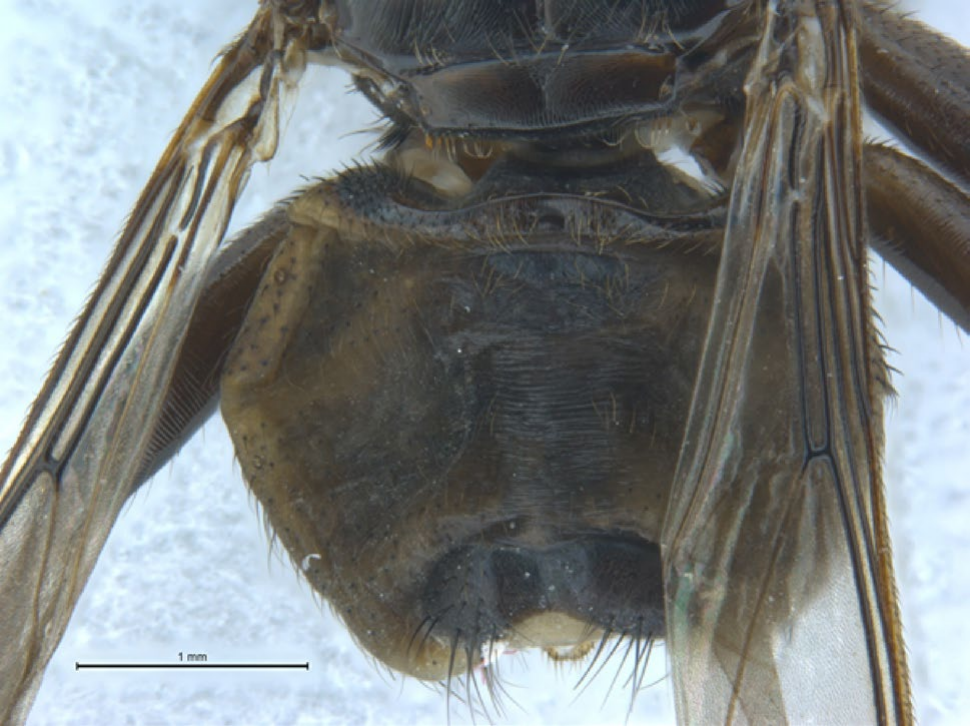
Photograph of the abdomen of the specimen of *Pseudolynchia garzettae* that was sequenced. The setae have faded during storage

Abdomen: (Fig. 4). Setae on the dorsal and ventral abdomen are uniform in colour, length and fineness and fairly evenly spaced, except for a row of longer setae in a row anterior to the urogenital area, which are slightly longer, and longer setae on the tergites.

Tergites 4 and 5 are absent. Tergite 6 is not interrupted.

**Fig. 5:**
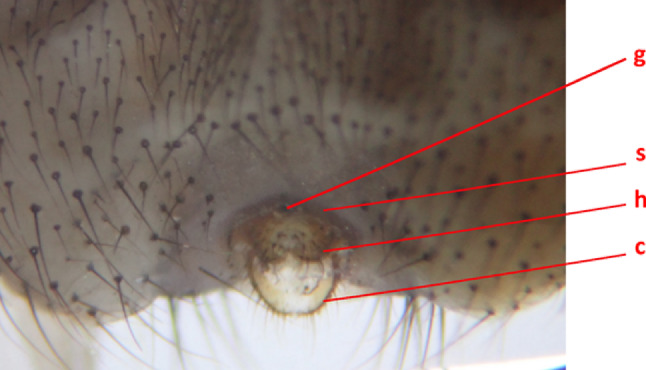
Ventral view of external genitalia of the sequenced specimen of *Pseudolynchia garzettae*: g, genital opening; s, sternite 8; h, hypoproct; c, cerci

Genitalia: (Fig. 5). The genitalia were not dissected. The terminalia are described with the fly on its back, positioned as for the photograph of the ventral habitus (Fig. 1b), using the terminology in section of the Manual of Nearctic Diptera describing the Hippoboscidae [25] after checking that these terms are up-to-date [7], with earlier terms listed to allow reference to older descriptions.

There is a sclerotised area immediately anterior to the genital opening (vulva) described as sternite 8 in the most recent works by Maa [25], but previously termed the anterior genital plate or ventral genital plate [25] and the pregenital plate [20]. Sternite 8’s structure is a shallow inverted V-shape. Maa described the pregenital plate as “elongate-triangular”, with the width at the posterior end twice or more that of the diameter of the largest setigerous papillae in its vicinity [20] as is seen in this specimen.

The hypoproct (infra-anal plate in earlier works, and pre-genital plate in one recent publication [19] lies immediately posterior to the genital opening. It is apparently composed of two lobes, each lateral to the midline, with no clear demarcation between them. The overall appearance is that of an inverted heart-shape, with a paler concave area in the centre, which has 8 setae on either side, mostly in the posterior part. Maa described these setae as “situated almost entirely in the posterior half” of the hypoproct [20].

The cerci (anal frame in earlier works) arise anteriorly from the lateral part of the hypoproct, curving to meet posteriorly, forming a ring that is covered in short setae of varying lengths.

There is no supra-anal plate, as expected in all *Pseudolynchia* species [22].

**Fig. 6:**
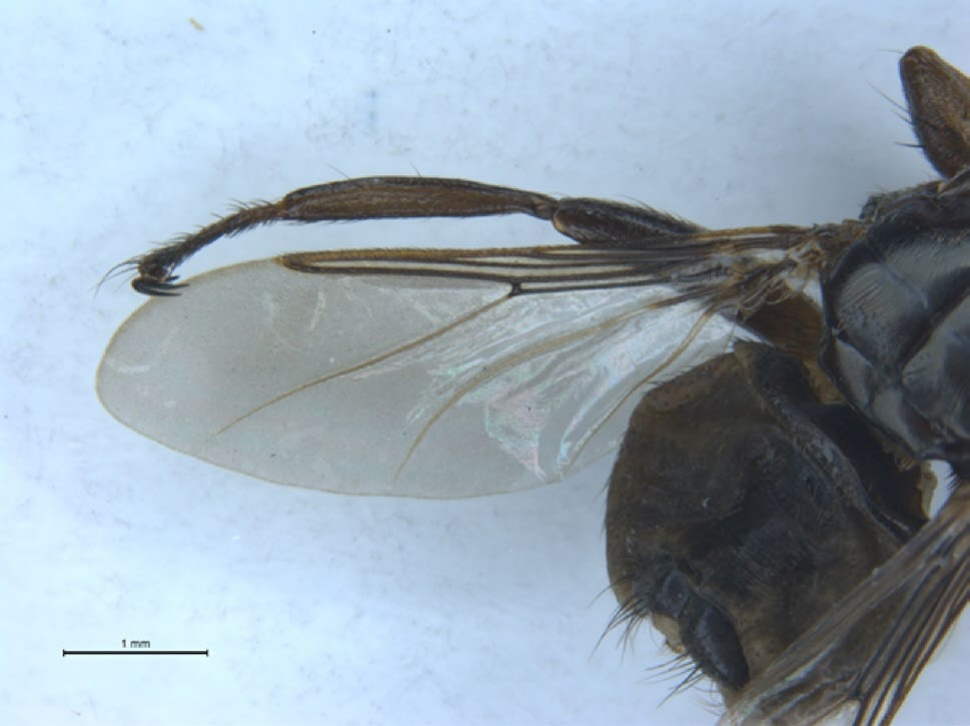
Photograph of the wing of *Pseudolynchia garzettae* that was sequenced

Wing: (Fig. 6): wing length 5.2 mm, falling within the stated range of 5–5.5 mm [20]. Only one crossvein, rm, is present. Cell 2a is narrower than the distance between veins rm and M3 + 4.

Microtrichia (setulae) cover the entire surface of cells 3r and 1 m, except for a bare area near the basal half of vein M3 + 4. Cell 2 m has a bare strip along vein cu + 1 A. Cell 2a is bare. The smaller wing cells, 1r, 2r and 1bc have microtrichia along their lengths which do not cover the whole surface of these cells.

**Fig. 7:**
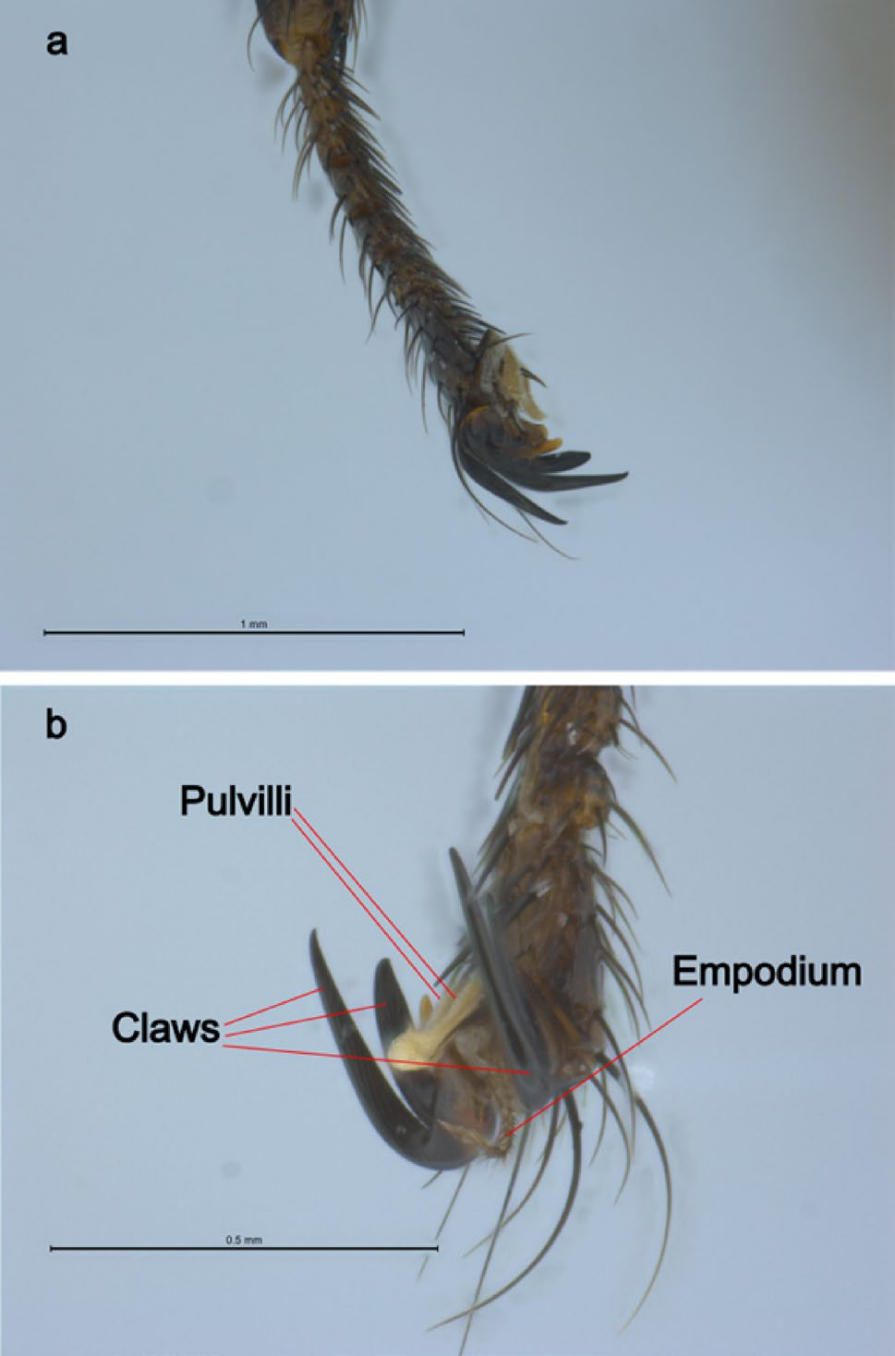
Photographs of the specimen of female *Pseudolynchia garzettae* that was sequenced. **a**, lateral view of right hind tarsus; **b**, ventral view of left hind leg to show pulvilli and empodium

Legs: (Fig. 7). As described by Rondani [33], all six legs are a uniform shade of dark brown.

Fore femur is uniformly covered in setae as described by Maa [22].

Hind tibia has 19 sensoria on the posterior surface – Maa described “ca. 18 sensoria” in this area [20]. Sensilla are also present in this region, as described by Maa, the number is not stated and the accompanying diagram is truncated before the point at which the sensilla stop [20].

Segment 1 of the hind tarsus, when seen from above, is nearly equal in length to segments 2 + 3. Sensilla are present on the lateral and medial margins of segment 1 of the hind tarsus, but are difficult to enumerate. Maa said segment 1 has “ca. 14 and 2 sensilla on “inner” and “outer” margins respectively” [20]. Hind tarsus, segment 2 is shorter than wide and almost symmetrical. Segments 3 and 4 are asymmetrical.

The stout black spines, on the inferior-medial surface of the tarsi segments 1–4, are as described by Maa [20], including the additional spine on segment 1 of the fore tarsus: fore leg, 2-1-1-0 spines, (on segment1, segment 2, segment 3 and segment 4 respectively), 2-1-1-1 on the middle leg and 2-2-2-2 on the hind leg. It should be noted however, that although the single spine on segment 2 of the middle leg tarsus is the same as that shown in Maa’s figure, it is much weaker than the other spines and hard to distinguish from the ordinary setae that have been omitted from Maa’s diagrams, and that it could be argued that the spine of the middle tarsus might be more accurately described as 2-0-1-1.

### Molecular and Phylogenetic Data

COXI sequences were available in NCBI GenBank for 19.3% (38/197) and full mitochondrial sequences 5.6% (11/197) of the species of Hippoboscidae listed in the checklist [8]. A range of other mostly partial sequences were available for a few Hippoboscid flies. These included some longer and overlapping sequences, which were described as “cytochrome oxidase subunit II (COII)”, “large subunit ribosomal RNA”, “small subunit ribosomal RNA gene”, “12s ribosomal RNA”, “16s ribosomal RNA”, “18s ribosomal RNA” and “28s ribosomal RNA”, “ribosomal RNA gene”, “internal transcribed spacer 2”, “cytochrome b”, “carbamoyl phosphate synthetase (CAD)”, “trisephosphate isomerase”, “anayl-tRNAsynthetase (AATS)”, “elongation factor-1 alpha (EF)” and “6-phosphogluconate dehydrogenase”.

DNA sequencing of the selected specimen of *P. garzettae* yielded a 662 bp long consensus sequence (NCBI GenBank accession number PQ740968). The closest match to the sequence found using the BLASTN function in GenBank was a 96% match with *Pseudolynchia canariensis*. The sequences from flies identified only to genus were all a 97.77% or greater match with *P. canariensis*, with all but one of the sequences being a greater than 99.5% match with *P. canariensis*.

A comparison in GenBank BLASTN with the closest match (*Pseudolynchia canariensis* isolate Pigeon Keds, Pigeon Louse Fly cytochrome c oxidase subunit I (COX1) gene, partial CDS; mitochondrial Sequence ID: OM073981.1) with CDS feature enabled, showed the new COX1 sequence for *Pseudolynchia garzettae* differed by 23 base pairs from the *P. canariensis* sequence. However, only two of these base substitutions in this short sequence have resulted in a change in the amino acid coded: alanine and valine in *P. garzettae* compared to threonine and methionine in *P. canariensis*. The sequence was a 100% match with a shorter, 313 bp sequence, recently reported for *P. garzettae* [19].

**Fig. 8:**
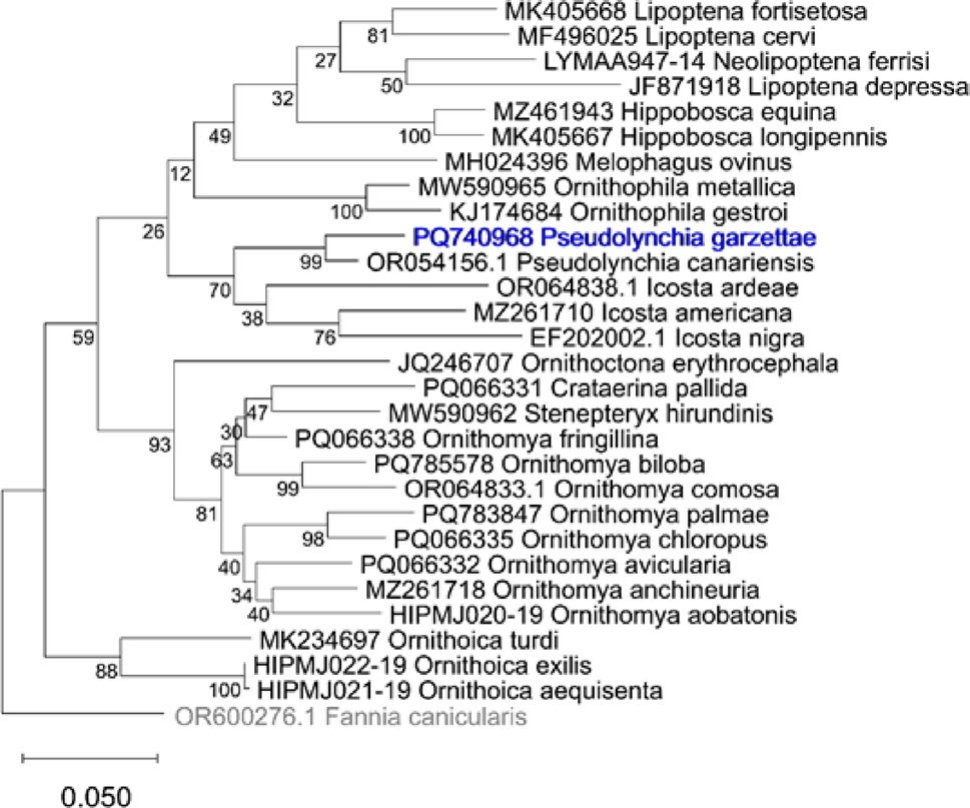
Phylogenetic tree of COXI sequences, showing the position of *Pseudolynchia garzettae* within the *Hippoboscidae*, with the Dipteran *Fannia canicularis* (*Linnaeus* 1758) as an outgroup. The scale bar indicates the branch length (number of substitutions per site between the nodes) and the numbers the bootstrap values of the nodes

**Fig. 9:**
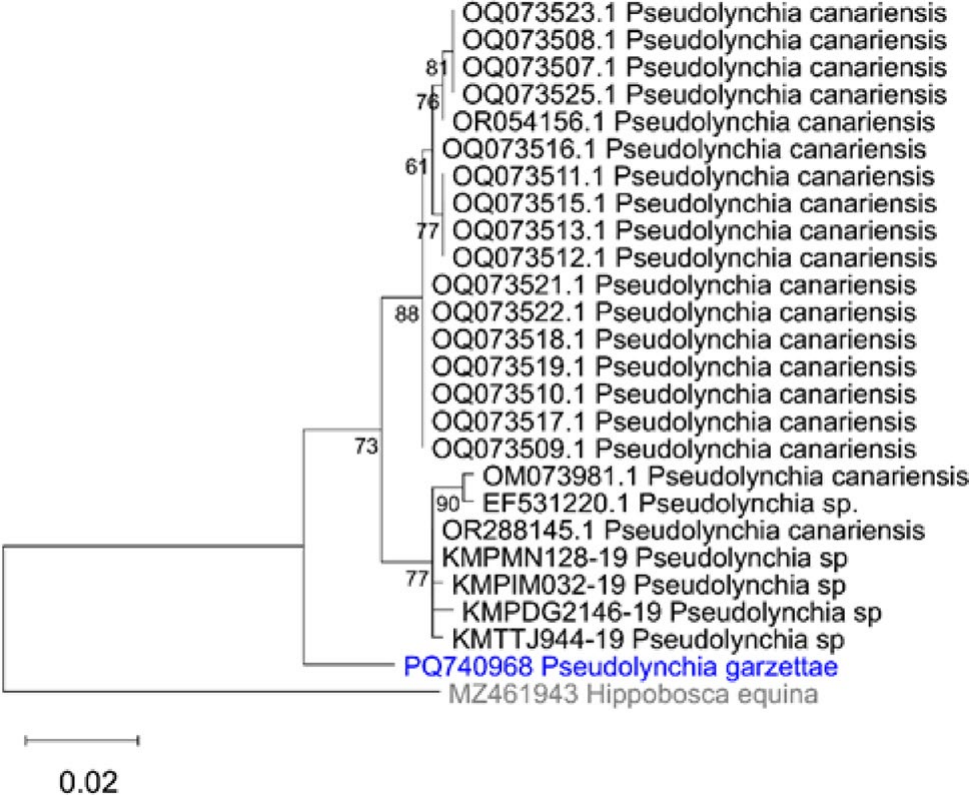
Phylogenetic tree of COXI sequences showing the position of *Pseudolynchia garzettae* within the genus *Pseudolynchia*, with *Hippobosca equina* *Linnaeus* 1758 as an outgroup. The scale bar indicates the branch length (number of substitutions per site between the nodes) and the numbers the bootstrap values of the nodes

Phylogenetic trees plotted in MEGA, using sequences from GenBank and BOLD, for the Hippoboscidae (Fig. 8) and for the genus *Pseudolynchia* (Fig. 9)) also show that *Pseudolynchia garzettae* is most closely related to *P. canariensis*, with strong support for the node (bootstrap value 99). The tree (Fig. 8) places the genus *Pseudolynchia* in close proximity to the genus *Icosta* (Speiser, 1905), which is large genus represented here by the species, *Icosta americana* (Leach, 1905), *Icosta ardeae* (Macquart, 1835), and *Icosta nigra*183 (Perty, 1833). However, the bootstrap value supporting this node is lower (70) and sequences of a suitable length for the other four species in the genus *Pseudolynchia*, and for most of the species in the genus *Icosta* were not available.

The second phylogenetic tree (Fig. 9) also shows that all of the sequences from specimens only identified to the level of the genus *Pseudolynchia* are *P. canariensis.*

This phylogenetic tree was produced from sequences obtained from the GenBank and BoldSystems websites and analysed in MEGA version 12 [16]. The analysis was run on five computing threads, for 1000 replicates using the standard bootstrapping setting [9] to add confidence limits to this maximum likelihood tree, produced using a nearest neighbour interchange (NNI) and uniform rates in the Tamura-Nei model [36] of nucleotide substitutions.

This phylogenetic tree was produced from sequences obtained from the GenBank and BoldSystems websites and analysed in MEGA version 12 [16]. The analysis was run on five computing threads, for 1000 replicates using the standard bootstrapping setting [9] to add confidence limits to this maximum likelihood tree, produced using a nearest neighbour interchange (NNI) and uniform rates in the Tamura-Nei model [36] of nucleotide substitutions.

## Discussion

The DNA sequencing using relatively short COXI sequences supports the current taxonomy that places *P. garzettae* in the genus *Pseudolynchia* with *P. canariensis*, and places the genus in close proximity to the genus *Icosta*. All of the other sequences from specimens only identified as *Pseudolynchia* sp. in GenBank and BOLD were confirmed as *Pseudolynchia canariensis*. No suitable length sequences were available for the other four species in the genus *Pseudolynchia*.

The morphological determination of species within the genus *Pseudolynchia* is complex. Maa reported a lack of distinct features useful in separating *P. garzettae* from other members of the genus and listed a range of features that have been used by various authors to distinguish the species and subsequently abandoned [20]. Often the suggested criteria ([20, 22]) are somewhat subjective, relying on performing comparison with other species, typically *P. canariensis*, to compare features such as overall body size, length of the femur, etc. Maa found the differences in genitalia between the species to be slight in both sexes [20]. Table 2 contains a summary of features that may be useful in identifying these species which do not rely on comparisons with other species, microscopes with high magnification – to count sensoria or sensilla – or a detailed knowledge of hippoboscid anatomy. The main structures of the genitalia have been added to Table 2 for completeness, despite being of little use in the genus *Pseudolynchia*.

**Table 2:** Comparison of *P. garzettae* with other species in the genus *Pseudolynchia*, compiled from various sources [19, 20, 22, 30], with an emphasis on characteristics which can be used without reference to other species or genital dissection. The main structures of the external genitalia are included for completeness but they are generally unhelpful in determining species in the genus *Pseudolynchia*. The features for *P. maai* marked * have been completed by extraction of details from the photographs that accompany the species description and may not agree with the authors’ statement in the description that “the two species are morphologically similar” [19] possibly due to individual variation between specimens

Characteristic	*Pseudolynchia garzettae* (Rondani, 1879)	*Pseudolynchia canariensis* (Macquart, 1839)	*Pseudolynchia maai* Lee & Obona, 2025	Pseudolynchia serratipes Maa, 1966	*Pseudolynchia brunnea* (Latreille, 1811)	*Pseudolynchia mistula* Maa, 1969
Distribution	Africa, Mediterranean, parts of Asia, increasingly common in Europe	All continents, except Antarctica	Singapore	New Guinea	North and South America	New Guinea
Host preference	Caprimulgidae, Strigidae	Columbiformes, a range of other species	Eastern Spotted Dove *Spilopelia chinensis* (Scopoli, 1786), Pink-necked Green Pigeon, *Treron vernans* (Linnaeus, 1771).	Orange- bellied Fruit Dove *Ptilinopus* *iozonus* G.R.Gray, 1858	Multiple species including *Caprimulgus* sp.	Quail
Wing length	5–5.5 mm long	4.5–7.5 mm	NOT STATED	6.5 mm	4.8–6 mm	5.2 mm
Wing microtrichia Cell 3r	Entirely covered	Entirely covered	NOT STATED* From images: small clear at proximal end of 3r and adjacent to distal part of vein R4 + 5	Entirely covered	Entirely covered	Entirely covered
Wing microtrichia Cell 1 m	Bare streak near basal half of vein M3 + 4 (usually)	Bare streak near basal half of vein M3 + 4 (usually)	NOT STATED* Image shows bare streak near vein M3 + 4, separated from the vein by a thin tract of microtrichia	No bare strip	Bare streak near vein M3 + 4	Bare streak near vein M3 + 4
Wing Microtrichia cell 2 m+1a	None on basal one fifth to half	None on basal one fifth to half	NOT STATED* Image shows none on basal one fifth to one half	Extreme base bare	Entirely covered	Entirely covered
Wing microtrichia cell 2a	Bare throughout	Bare throughout	NOT STATED* Image shows it is entirely bare	Bare throughout	Bare throughout	Bare throughout
Prescutellar setae	Black	Pale	Long, pale, fine only	Yellow	Yellow	Black, robust
Posterior border of scutellum	Very gently curved	Straight	Straight	Very gently curved	Very gently curved	Practically straight
Finger-like processes on posterior margin of scutellum	Long, mostly not more slender than scutellar setae	Long, mostly not more slender than scutellar setae	NOT STATED	Vestigial, hardly longer than wide, with diameters only ca. 1/2 that of scutellar setae	Long, mostly not more slender than scutellar setae	Well-developed
Scutellum ratio of length: width between scutellar setae	1:3	1:4	NOT STATED* 1:4 from images	2:5	1:3	1:3
Height of segment 1 of hind tarsus equal to	Almost equal so segments 2 + 3	About the same as segments 2 + 3	NOT STATED	Slightly longer than segments 2 + 3+4	Slightly shorter than segments 2 + 3	Slightly longer than 2 + 3
Segment 4 of hind tarsus	Very asymmetrical	Very asymmetrical	NOT STATED	Virtually symmetrical	Distinctly asymmetrical	Distinctly asymmetrical
Stout apical spines inferior medial surface of segments 1–4 of fore tarsus	Female: 1(+ 1minor)-1-1-0 Male: 1-0-1-0	1-0-1-0	NOT STATED	1-0-1-0	1-0-1-0 (male not described)	1-0-1-0
Stout apical spines segments 1–4 of middle tarsus	Female: 2-1-1-1 Male: 1-0-1-1	Female: 2-1-1-0 Male: 1(+1 minor)-0(+1 minor)-1-0	NOT STATED	1-1-1-0	1-1-2-0 (male not described)	1-?-?-?
Sternite 8 (female) Also known as: ventral genital plate, anterior genital plate, pre-genital plate.	Elongate triangular (inverted shallow V-shape), width at posterior end 2x or more diameter of largest setigerous papillae in its vicinity.	As in *P. garzettae*	(“Distinctly sclerotised on the sides, leaving a pale strip in the centre” this would, however, appear to be a description of the hypoproct)	Longitudinally linear, width not greater than diameter of largest setigerous papillae in its vicinity.	Similar to *P. canariensis*	Pregenital plate longitudinally linear, hardly pigmented and sclerotized.
Hypoproct (female) (Infra-anal plate)	Setae generally largely situated on the posterior ½ of the plate	Setae generally largely situated on the posterior ½ of the plate	Distinctly sclerotised on the sides, leaving a pale strip in the centre	Setae almost entirely situated on the posterior half of the plate	Setae paler and finer than in P. canariensis, largely situated on the posterior ½ of that plate	Not described
Aedeagus (male)	Profile slightly more slender, less curved, and longer than *P. canariensis.*	Profile slightly more robust, more curved, and shorter than *P. garzettae.*	“Morphologically similar” to *P. canariensis.*	Male of species unknown	Not described	Male of species unknown
Paramere (male)	Profile slightly more slender and less curved than *P. canariensis.*	Profile slightly more robust and more curved than *P. garzettae.*	“Morphologically similar” to *P. canariensis.*	Male of species unknown	Not described	Male of species unknown

Despite using only a single relatively short DNA sequence, the phylogenetic tree produced from sequences of unidentified flies in the genus *Pseudolynchia* obtained from GenBank and BOLD gives a similar result to that obtained by Sochova et al., [34]. Using COXI, elongation factor-1 alpha and 16 S ribosomal RNA genes sequences, they produced a phylogeny showing that all of their specimens of flies identified only as *Pseudolynchia sp.*, obtained from South Africa, fell within a single clade with *Pseudolynchia canariensis* [34]. Further analysis of the data by another group of researchers confirmed this result [35]. Unfortunately, the COXI sequences from these studies were only 208–213 base pairs long so were excluded from the phylogeny in this study, and the flies were destructively sampled so are not available for re-examination.

Further sequencing, using longer sequences, from accurately identified additional species in the genera *Icosta* and *Pseudolynchia* will be necessary to determine if these two genera are monophyletic, and their relationship to the other genera, *Microlynchia*,* Olfersia* and *Phthona*, that Maa grouped in the “Icosta complex” [23]. Maa found no clear-cut morphological dividing line between the two genera, apart from the presence or absence of a short, colourless vein 1 m [23] and it may be that *Pseudolynchia* may be better considered a subgenus of *Icosta*, in line with the subgenera, *Ardmoeca*, *Gypoeca*, *Icosta*, *Ornithoponus*, and *Rhyponotum*, listed by Maa [8, 23] but any taxonomic revision should await the results of further sequencing. However, many of the species necessary to produce a comprehensive phylogeny are not only unsequenced but are rarely recorded.

This additional sequence may help with the identification of some louse flies by “DNA barcoding”. It may be particularly important as this species is increasingly being found in Europe ([18, 29, 31, 38]; Evens & Wawman, unpublished data) and may also be extending its range at its northeastern edge. It will also help to determine the relationship between species in the genus *Pseudolynchia* and be useful the designation of potential new species of louse flies, although the use of longer sequences, and/or whole genome sequencing, may be needed to explore the relationships of species beyond the of genus.

## Data Availability

The DNA sequence is available in NCBI GenBank with accession number PQ740968.The louse fly is available in the collection of Oxford University Museum of Natural History, Oxford, UK, accession number ENT-OUMNH-2025-042-0001.
